# Exosomal miR-200c and miR-141 as cerebrospinal fluid biopsy biomarkers for the response to chemotherapy in primary central nervous system lymphoma

**DOI:** 10.1007/s12672-023-00812-1

**Published:** 2023-11-16

**Authors:** Yao Hu, Qingyun Zhang, Zhiyuan Wu, Kun Chen, Xiao Xu, Weizhe Ma, Bobin Chen, Limin Jin, Ming Guan

**Affiliations:** 1 Department of Laboratory Medicine, Huashan Hospital, Shanghai Medical College, Fudan University, Shanghai, 200040 China; 2 Department of Central Laboratory, Huashan Hospital, Shanghai Medical College, Fudan University, Shanghai, 200040 China; 3 Department of Hematology, Huashan Hospital, Shanghai Medical College, Fudan University, 200040 Shanghai, China; 4 Department of Laboratory Medicine, Jiaxing Hospital of Traditional Chinese Medicine, Zhejiang Chinese Medical University, Jiaxing, 314001 Zhejiang China

**Keywords:** Primary central nervous system lymphoma, Cerebrospinal fluid, Exosome, microRNA, Chemotherapy

## Abstract

**Background:**

To improve early diagnosis and chemotherapy efficacy monitoring in primary central nervous system lymphoma (PCNSL), cerebrospinal fluid (CSF) exosomal microRNA (miRNA) studies were performed.

**Method:**

Small RNA sequencing was performed to identify candidate exosomal miRNAs as CSF biopsy biomarkers from two patients with de novo PCNSL and two patients in remission after chemotherapy. miR-200c and miR-141 expression in CSF exosomes was further validated using relative quantitative real-time polymerase chain reaction in patients with PCNSL (n = 20), patients with other neurological diseases (n = 10), and normal subjects (n = 10). Receiver operating characteristic (ROC) curve analyses of miR-200c and miR-141 in the diagnosis and prediction of chemotherapy efficacy in PCNSL were performed in patients treated with methotrexate. Additionally, bioinformatics tools were utilized to predict the potential targets of miR-200c and miR-141.

**Results:**

Exosomal miR-200c and miR-141 levels in CSF from patients with PCNSL were significantly lower than those in control subjects. Importantly, miR-200c and miR-141 were upregulated in patients with PCNSL after chemotherapy (P = 0.002). There was a significant correlation between the levels of miR-141 and IL-10 in CSF (P = 0.04). The combination of miR-200c and miR-141 yielded an area under the ROC curve of 0.761 for distinguishing PCNSL with sensitivity and specificity of 60.0% and 96.7%, respectively. The potential target genes of miR-200c and miR-141 in PCNSL included ATP1B3, DYNC1H1, MATR3, NUCKS1, ZNF638, NUDT4, RCN2, GNPDA1, ZBTB38, and DOLK.

**Conclusion:**

Collectively, miR-200c and miR-141 are likely to be upregulated in CSF exosomes after chemotherapy in patients with PCNSL, highlighting their potential as reliable liquid biopsy biomarkers for PCNSL diagnosis and chemotherapy efficacy monitoring.

**Supplementary Information:**

The online version contains supplementary material available at 10.1007/s12672-023-00812-1.

## Introduction

Primary central nervous system lymphoma (PCNSL) emerges directly from the central nervous system and progresses rapidly with poor prognosis [1]. Diffuse large B-cell lymphoma (DLBCL), the most common PCNSL subtype, is a group of heterogeneous tumors [2]. The incidence of PCNSL is steadily increasing as the population ages [3–5]. In the United States, the incidence increased from 0.1/100,000 to 0.5/100,000 with an average annual percent change of 5.3% from 1975 to 2017 [6]. The current standard of care for patients with PCNSL is high-dose methotrexate-containing combination chemotherapy. The 5-year survival rate for elderly patients was approximately 30% in the 2010s [7]. Although treatment improves the median survival to 40–60 months, 50% of patients with PCNSL experience relapse after treatment, and the median survival is only 4–12 months [8]. Meanwhile, 15% of patients have refractory disease, and their overall prognosis is typically worse [9, 10]. Imaging technologies and cerebrospinal fluid (CSF) cytology are the gold standards for the diagnosis of PCNSL. But these tools have low sensitivity and specificity with respect to disease staging, and lack power for monitoring treatment responses, in addition to being cumbersome and expensive [11]. To enable early diagnosis and chemotherapy monitoring in PCNSL, it is essential to identify sensitive biomarkers.

CSF liquid biopsy biomarkers might assist in the diagnosis of PCNSL [12]. In our previous study, we demonstrated that interleukin 10 and free light chains in CSF can potentially serve as protein markers for diagnosis and chemotherapy efficacy monitoring in PCNSL [13, 14]. Exosomes exist and circulate in CSF, enclosing cytosolic proteins and nucleic acids, especially microRNAs (miRNAs). Because miRNAs enclosed in exosomes are commonly protected from degradation by RNases, and it was speculated that they are reliable prognosis predictors associated with cancer immunity [15, 16]. A large number of miRNAs were identified as markers for both B-cell differentiation stage and malignant transformation [17]. Recently, several miRNAs were detected not only in tumor cell but also in plasma of patients with NHL by quantitative real-time polymerase chain reaction (qRT-PCR), and several miRNAs are abnormally regulated in DLBCL, such as miR-141 [18–20]. Although the evaluation of exosomal miRNAs could provide important information for diagnosis and monitoring treatment efficacy in DLBCL [21, 22], research regarding exosomal miRNAs from CSF in patients with PCNSL remains scarce.

This exploratory study analyzed CSF-derived exosomal miRNAs for biomarker discovery in PCNSL. Candidates were identified by miRNA sequencing in CSF samples from patients with PCNSL and further validated in independent CSF samples. Two members of the miR-200 family (miR-200c and miR-141) derived from CSF exosomes were identified as promising liquid biopsy biomarkers for improving early diagnosis and monitoring chemotherapy efficacy in PCNSL.

## Materials and methods

### Participants

From January 2020 to March 2022, 20 PCNSL patients and 10 patients with various intracranial lesions of non-tumor etiology were included in the current study at Huashan Hospital of Fudan University. In addition, 10 subjects with no recent history of symptoms of neurological disease or head trauma served as normal controls. Besides the clinical symptoms, all patients underwent brain magnetic resonance imaging as part of the diagnostic work-up prior to lumbar puncture. Demographic and clinical data were recorded from the electronic medical records.

The enrolled patients with PCNSL were classified according to the World Health Organization classification of tumors of hematopoietic and lymphoid tissues [23]. All patients underwent histopathologic examination, and HIV negativity were confirmed. Among the 20 PCNSL samples, 8 with initially diagnosed PCNSL (de novo disease) comprised the pre-treatment group, and 12 comprised the post-treatment group. Comparison of pre-treatment and post-treatment after high-dose methotrexate (MTX) was made in 8 PCNSL patients, nevertheless, 4 PCNSL patients who entered remission were added into the post-treatment group.

### Preparation of CSF

CSF was collected from each patient via lumbar puncture during surgery and centrifuged at 2000×*g* for 10 min to remove cells and cell fragments. CSF samples were immediately stored at − 80 ℃ until further analyses. All samples were handled according to the standardized laboratory procedures.

### Exosome isolation and characterization

The fractionation and purification of exosomes from CSF (10 mL) were conducted through standard centrifugation steps as previously described [24]. Briefly, the CSF samples were centrifuged at 3000×*g* for 10 min at 4 ℃ to remove large fragments. The supernatant was carefully transferred to a fresh tube, which was centrifuged at 100,000×*g* for 70 min at 4 ℃, and the supernatant was removed. Then, the precipitate was resuspended in phosphate-buffered saline (PBS, pH 7.2–7.4), and the previous step of ultracentrifugation was repeated. Finally, the precipitate was resuspended in 400 µL of PBS and stored at − 80 ℃. Isolated exosomes were identified by nanoparticle tracking analysis (NTA), and transmission electron microscopy (TEM) (Additional file 1: Supplementary methods).

### Exosomal RNA extraction and RNA quality control

Extraction and purification of CSF exosome total RNA were performed using an miRNeasy Advanced Kit (Qiagen, cat. No. 217204) according to the manufacturer’s recommendations. RNA concentration and purity were evaluated by the RNA Nano 6000 Assay Kit of the Agilent Bioanalyzer 2100 System (Agilent Technologies, CA, USA).

### Exosomal miRNA-seq library preparation and next-generation sequencing (NGS)

Samples from two patients with PCNSL at initial diagnosis and two patients in remission after chemotherapy were selected for RNA profiling of CSF exosomes by NGS. High throughput sequencing was performed by EchoBiotech Co. Ltd. (Beijing, P. R. China). Sequencing libraries were generated using a QIAseq miRNA Library Kit (Qiagen, Frederick, MD, USA) following the kit instructions. The library quality was assessed using the Agilent Bioanalyzer 2100 (AgilentTechnologies, USA). The library preparations were sequenced on an Illumina NovaSeq 6000 platform, and paired-end reads were generated at EchoBiotech Co. Ltd. Finally, miRNA sequencing data and differential expression analysis were performed. See Additional file 1: Supplementary methods for details.

### qRT-PCR quantification of miRNA

To validate the expression of two miRNAs (miR-200c and miR-141), qRT-PCR was performed in the cohort of eight patients with newly diagnosed PCNSL at initial diagnosis, 12 patients with PCNSL in remission after chemotherapy, 10 patients with other neurological diseases, and 10 normal subjects. U6 was used as the endogenous control. Briefly, total RNA was extracted from exosomes extracted from 4.0 mL of CSF and purified from the exosomes using an miRNeasy Advanced Kit (Qiagen) following the kit instructions and then reverse-transcribed to synthesize cDNA using a PrimeScript™ RT reagent Kit (Perfect Real Time) (TAKARA, RR037A). The expression of miR-200c and miR-141 was detected by a TaqMan ® probe using real-time qPCR and normalized according to the expression of U6 small nucleolar RNA. Two microliters of cDNA were used as the template for each round of PCR. The primer sequences used in qRT-PCR are listed in Table S1. All experiments were repeated in triplicate wells. The relative expression of miR-200c and miR-141 was calculated using the 2^−ΔΔCt^ method as follow: ΔCt = Ct (miRNA) - Ct (U6) and Δ ΔCt = ΔCt (miRNA) - MAX ΔCt (miRNA).

### Bioinformatics analysis

For functional analyses of miR-200c and miR-141, Gene Ontology term and Kyoto Encyclopedia of Genes and Genomes pathway enrichment analysis was performed using the R package clusterProfiler (ver. 4.0). To investigate the correlations between miRNA expression and the prognosis of PCNSL, the target genes of miR-200c and miR-141 were predicted and analyzed using the multiMiR package ®, and the target genes predicted by at least three databases (software) or verified by at least one experiment, were selected as the prediction results. Based on the data of Lymphoid Neoplasm Diffuse Large B-cell Lymphoma (DLBCL) in the GEPIA2 database (http://GEPIA2.cancer-pku.cn/#index), the top 500 most differential survival genes that significantly affected overall survival (OS) and disease-free survival (DFS) were obtained to find target genes significantly related to patient survival.

### Statistical analyses

Descriptive and comparative statistical analyses were performed using SPSS (ver. 17.0; SPSS Inc.), and the charts were created using GraphPad Prism V6 for Windows. Continuous data were expressed as the median (interquartile range, IQR). The Mann–Whitney U test was used to compare the expression of miR-200c and miR-141 between different groups. Fisher’s exact test were used to determine the correlations between miR-200c or miR-141 expression and the clinical parameters of the subjects. The area under the receiver operator characteristic (ROC) curve (AUC) was calculated to explore the diagnostic role of miR-200c and miR-141. Two-sided P < 0.05 was considered statistically significant.

## Results

### Study participants’ characteristics

In total, 20 patients with PCNSL were recruited, including eight patients with de novo disease and 12 patients who achieved remission after chemotherapy. All patients were DLBCL. The median patient age was 65 years, and the cohort included seven women and 13 men. Meanwhile, 25% (5/20) patients with PCNSL had multiple primary intracranial lesions (≥ 2), 75% (15/20) had deep lesions (including the basal ganglia, corpus callosum, and periventricular area), and 30% (6/20) had leptomeningeal involvement at the initial diagnosis or during treatment. Ten patients (median age, 41 years) with various neurological diseases of non-tumor etiology (cranial/peripheral palsy, non-inflammatory neurological disease, headache, or sensory disturbances) were enrolled as disease controls. Ten health individuals (median age, 52 years) were enrolled as normal controls (four women and six men). The gender and age characteristics of the study population are summarized in Table 1.

**Table 1:** General information of the study population

Characteristics	PCNSL (n = 20)	Other neurological diseases (n = 10)	Controls (n = 10)	P value
Gender				0.69
Female, n (%)	7 (35%)	5 (50%)	4 (40%)	
Male, n (%)	13 (65%)	5 (50%)	6 (60%)	
Age, median (IQR)	65 (57, 74)	41 (36, 54)	52 (38, 62)	0.66

### Identification of CSF exosomal miRNA

The morphology of CSF exosomes was observed by TEM, as presented in Figure S1A. Using a particle size analyzer, we demonstrated that the width of exosomes ranged from 85 to 175 nm (Figure S1).

**Fig. 1:**
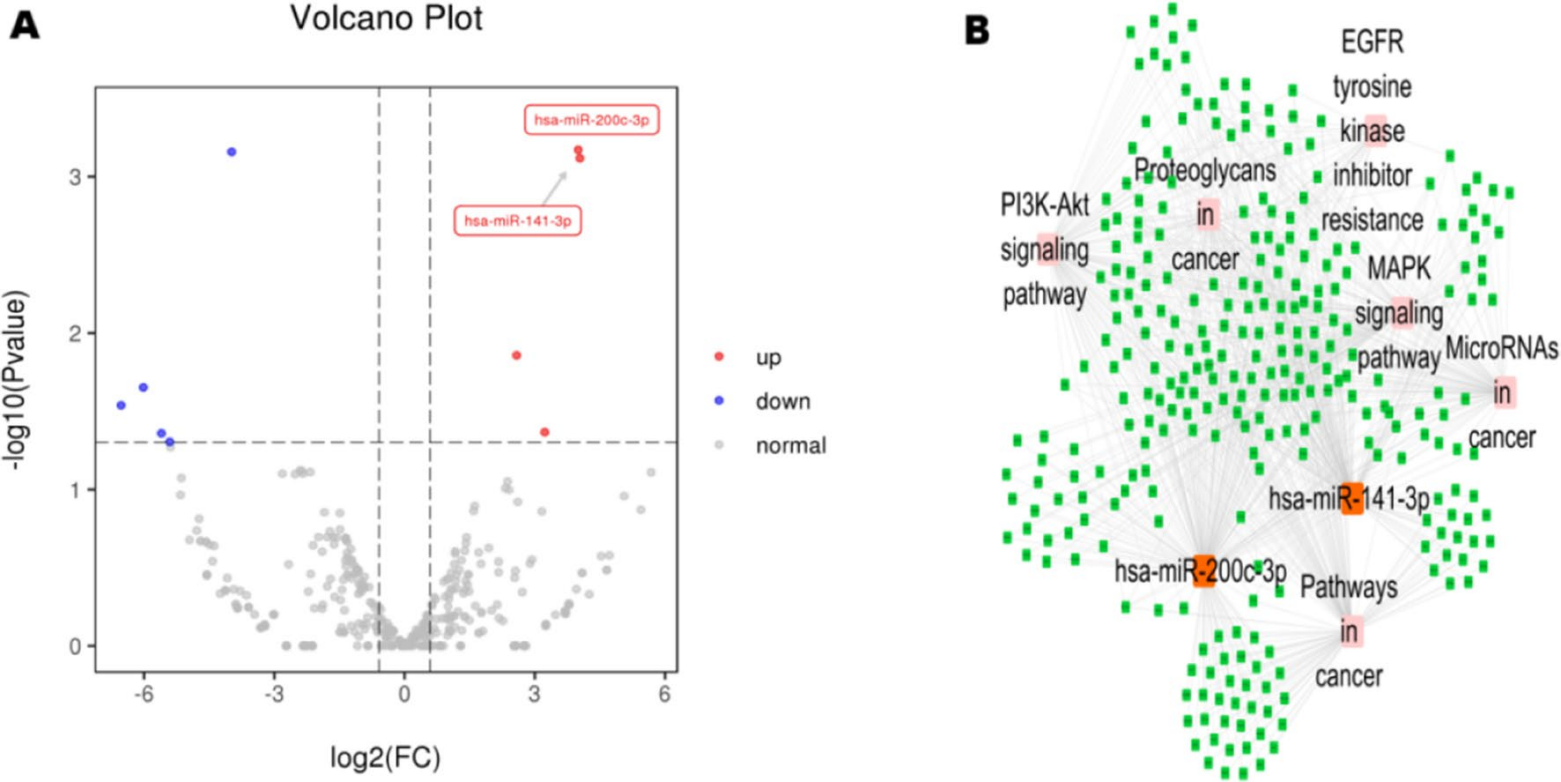
**A** Volcano plot of miR-200c and miR-141 expression in CSF-derived exosomes between patients with de novo PCNSL diagnosis and patients with previously treated PCNSL. **B** The interaction network diagram of miR-200c and miR-141

miRNA sequencing was then performed using CSF-derived exosomes from two patients with de novo PCNSL and two patients in remission after chemotherapy. On average, approximately 21 million reads were generated in each library, and 337 exosomal miRNAs were detected in each sample (Table S2). Nine miRNAs were differentially expressed between the two groups (P < 0.01, Table S3). Via intersection with the NCBI Gene Expression Omnibus (GSE28090), two miRNAs, namely miR-200c and miR-141, which were differentially expressed between cancer and normal samples and mainly concentrated in some cancer signal pathways, were chosen for further validation (Fig. 1).

### Validation of CSF exosomal miR-200c and miR-141 by qRT-PCR

**Fig. 2:**
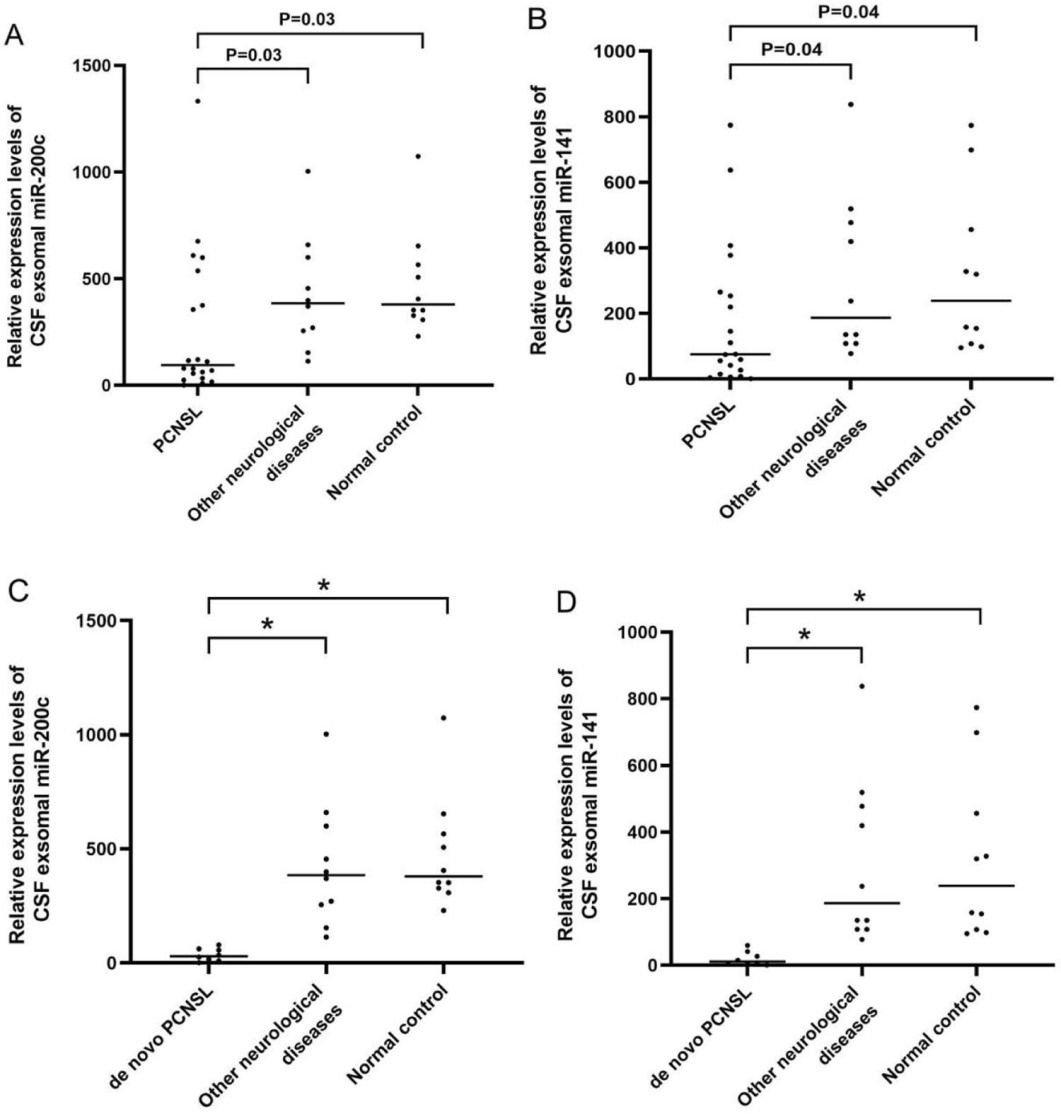
Comparison of the expression of miR-200c (**A**) and miR-141 (**B**) in CSF exosomes in the study population. The relative expression of miR-200c (**C**) and miR-141 (**D**) significantly differed between the de novo PCNSL and control groups. The Mann–Whitney U test was used for analysis (^*^P < 0.001).

Compared with the findings in the control group, the relative expression of miR-200c and miR-141 in CSF exosomes from patients with PCNSL was significantly decreased (P < 0.05, Fig. 2A, B). However, there was no significant difference in miR-200c and miR-141 expression in CSF exosomes between patients with other neurological diseases and normal controls (P > 0.05). Importantly, the same expression profile was discovered in CSF exosomes from patients with newly diagnosed PCNSL (P < 0.001, Fig. 2C, D). Thus, the results indicated the exosomal miR-200c and miR-141 in CSF were useful for discriminating patients with PCNSL from controls.

### Correlation between miR-200c and miR-141 expression and the clinical characteristics of patients with PCNSL

Further investigation focused on the correlation between miR-200c and miR-141 expression in CSF exosomes and the clinical characteristics of patients with PCNSL, as presented in Tables 2, 3. Patients with PCNSL were divided into high and low expression groups according to the median levels of miR-200c and miR-141. The level of miR-200c in CSF exosomes was associated with CSF IL-10 levels (P = 0.03) but not gender, age, the number of lesions, the CSF cell count, CSF cytology, and CSF protein levels (P > 0.05). In addition, our study found that only the level of IL-10 in CSF of PCNSL patients after treatment was significantly lower than that of de novo PCNSL patients (data not shown). However, no examined clinical factors were associated with miR-141 levels (P > 0.05).

**Table 2:** Correlation between the expression of miR-200c in CSF exosomes and the clinical characteristics of patients with PCNSL

Clinical parameters	Expression of miR-200c		P value
	Low, n = 7	High, n = 13	
Gender			
Female	3	4	0.65
Male	4	9	
Age			
≤ 65	2	3	0.59
> 65	5	10	
Lesions			
Single	5	10	0.59
Multiple	2	3	
CSF cell count (/µL)			
≤ 5	3	6	0.63
> 5	4	7	
CSF cytology (tumor cell)			
Negative	4	8	0.61
Positive	3	5	
CSF protein (mg/L)			
≤ 600	3	6	0.63
> 600	4	7	
CSF IL-10 (pg/mL)			
≤ 45	3	12	0.03
> 45	4	1	

**Table 3:** Correlation between the expression of miR-141 in CSF exosomes and the clinical characteristics of patients with PCNSL

Clinical parameters	Expression of miR-141c		P value
	Low, n = 9	High, n = 11	
Gender			
Female	3	4	0.63
Male	6	7	
Age			
≤ 65	2	3	0.60
> 65	7	8	
Lesions			
Single	8	7	0.22
Multiple	1	4	
CSF cell count (/µL)			
≤ 5	4	6	0.66
> 5	5	5	
CSF cytology (tumor cell)			
Negative	6	7	0.63
Positive	3	4	
CSF protein (mg/L)			
≤ 600	4	6	0.66
> 600	5	5	
CSF IL-10 (pg/mL)			
≤ 45	5	7	0.48
> 45	4	4	

### Evaluation of the diagnostic value of miR-200c and miR-141 expression in CSF exosomes from patients with PCNSL

**Fig. 3:**
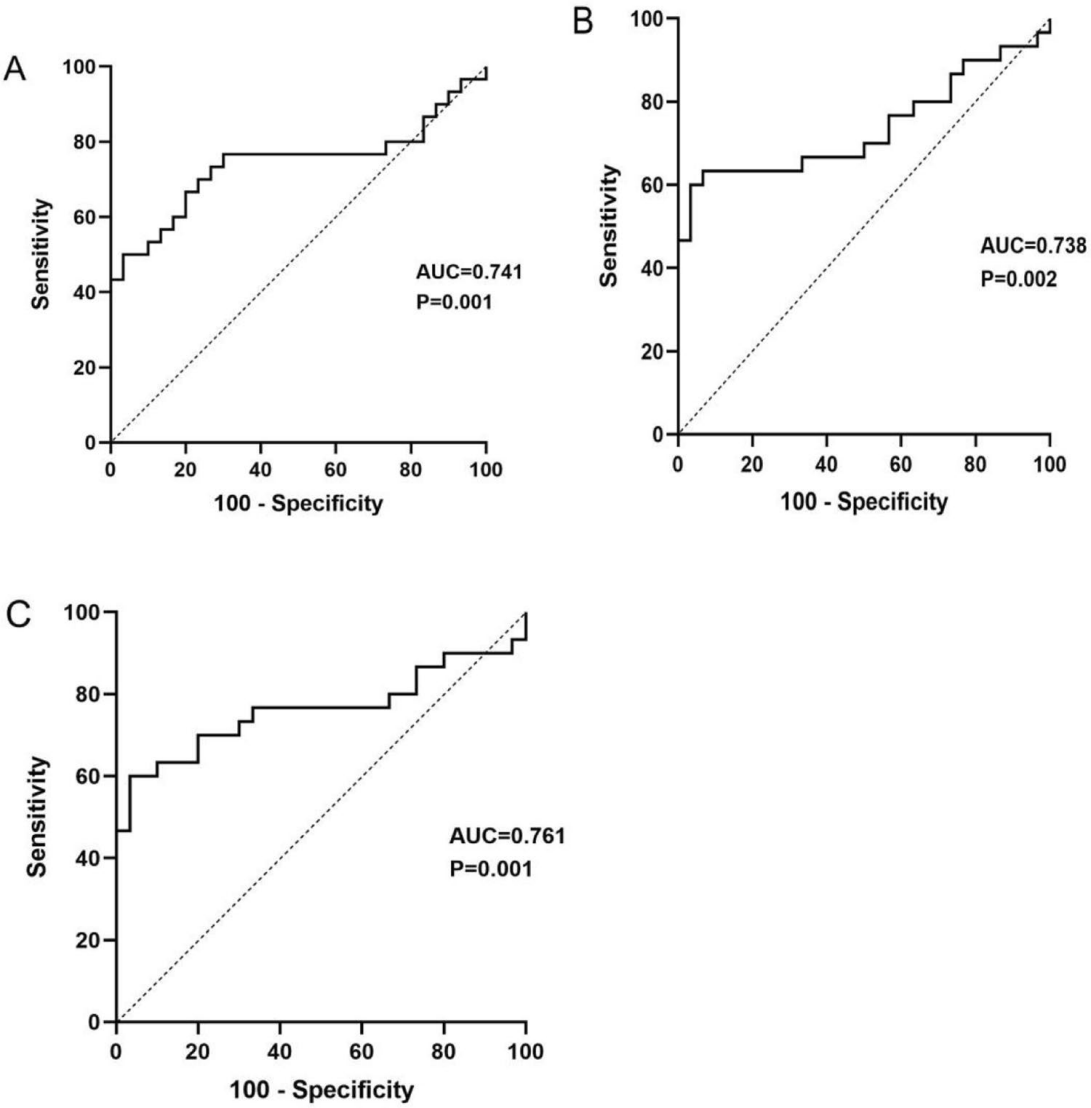
ROC curve analysis of CSF exosomal miR-200c (**A**), miR-141 (**B**), and miR-200c + miR-141 (**C**) for diagnosing PCNSL

ROC curves were drawn to determine the diagnostic performance of miR-200c and miR-141 levels in CSF exosomes from patients with PCNSL compared with the findings in 20 controls. As presented in Fig. 3A, the AUC of miR-200c for PCNSL diagnosis was 0.741 [95% confidence interval (CI) 0.606–0.876, sensitivity = 70.0%, specificity = 76.7%]. The AUC of miR-141 was 0.738 (95% CI 0.603–0.872, sensitivity = 60.0%, specificity = 96.7%). Intriguingly, the ROC curve of the combination of miR-200c and miR-141 for PCNSL diagnosis was drawn (Fig. 3C), and the most robust capacity of the combination for discriminating PCNSL was demonstration (AUC = 0.761, 95% CI 0.629–0.893, sensitivity = 60.0%, specificity = 96.7%).

### Clinical value of CSF exosomal miR-200c and miR-141 for monitoring chemotherapy efficacy in patients with PCNSL

**Fig. 4:**
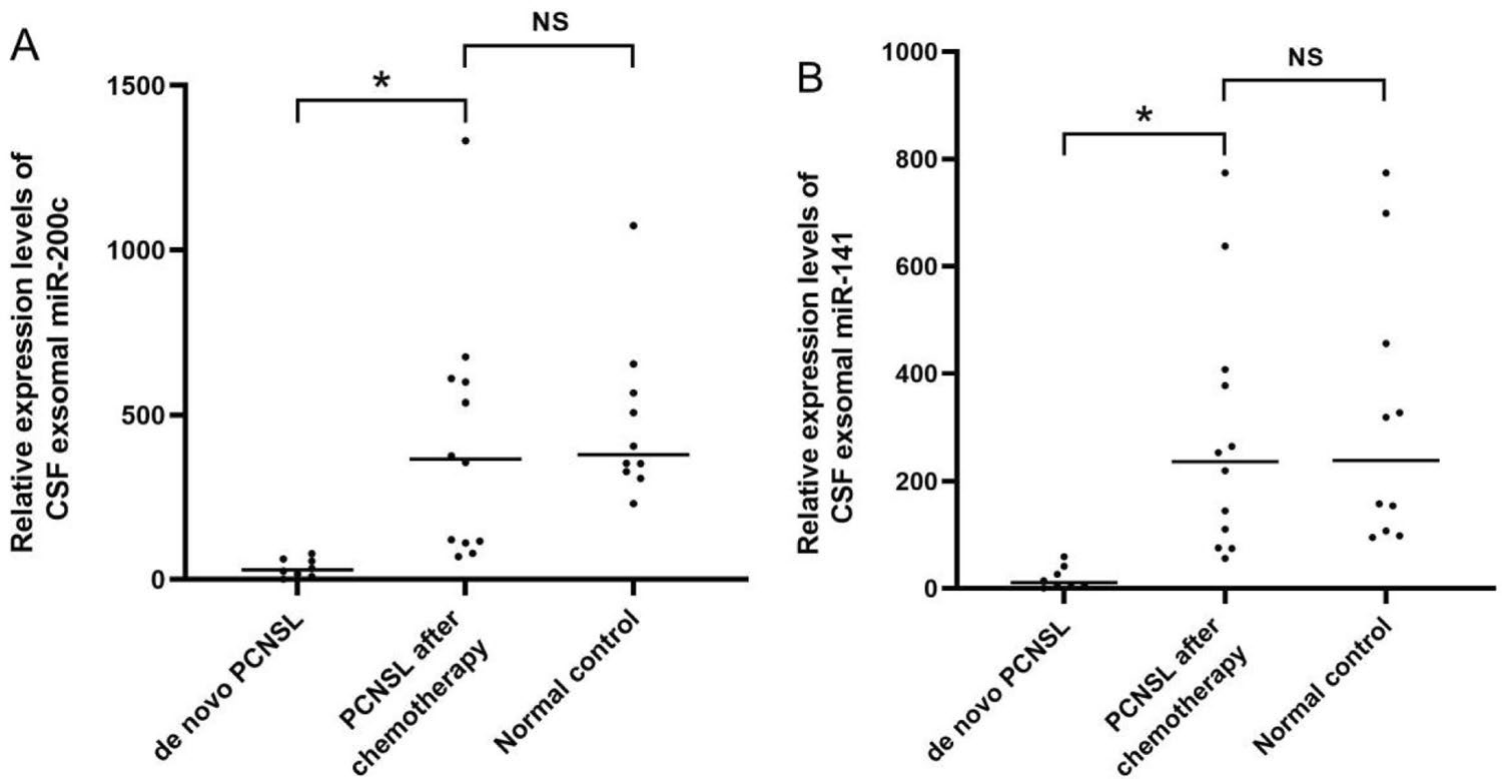
Changes of CSF exosomal miR-200c and miR-141 expression following chemotherapy. The relative expression of miR-200c (**A**) and miR-141 (**B**) was significantly increased in patients with PCNSL after chemotherapy. The Mann–Whitney U test was used for analysis (^*^P < 0.001; NS: not significant)

**Fig. 5:**
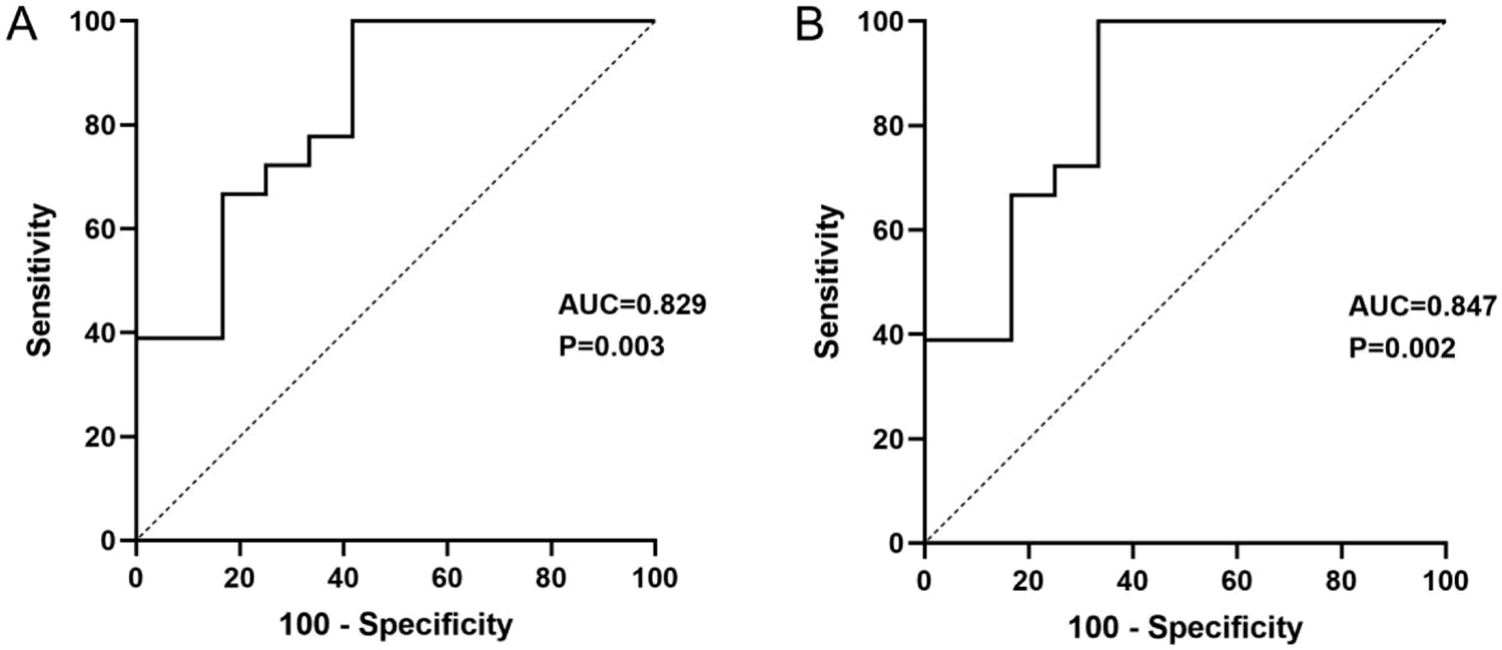
ROC curve analyses of miR-200c (**A**) and miR-141 (**B**) for predicting the response to MTX treatment in patients with PCNSL

To explore the potential utility of miR-200c and miR-141 for monitoring the efficacy of chemotherapy, the expression of miR-200c and miR-141 in CSF exosomes was compared between de novo PCNSL and PCNSL in remission after treatment with MTX. The characteristics of these patients are presented in Table 4. CSF exosomal miR-200c and miR-141 were distinctly upregulated in patients with PCNSL who received MTX (Fig. 4). Thus, miR-200c and miR-141 could be predictors of therapeutic response in patients with PCNSL treated with MTX. The data were further supported by ROC analyses, which revealed that CSF exosomal miR-200c (Fig. 5A) and miR-141 (Fig. 5B) were predictive of treatment response in patients with in PCNSL after chemotherapy (AUCs of 0.829 and 0.847, respectively).

**Table 4:** Comparison of CSF miRNA levels between pre-treatment and post-treatment patients

	Pre-treatment PCNSL	Post treatment PCNSL	P value
	n = 8	n = 12	
Gender			0.61
Female, n (%)	3 (37.5)	4 (33.3)	
Male, n (%)	5 (62.5)	8 (66.7)	
Ages, median (IQR)	64 (49, 72)	67 (59, 76)	0.65
miRNA relative expression			
miR-200c, median (IQR)	29.47 (11.48, 60.75)	365.61 (112.73, 606.88)	< 0.001
miR-141, median (IQR)	10.97 (4.56, 37.96)	236.47 (84.35, 399.79)	< 0.001

### Exploration of miR-200c and miR-141 target genes

In total, 2808 target genes of miR-200c or miR-141 were predicted, of which 1912 genes were predicted targets of miR-200c, 1274 genes were predicted targets of miR-141, and 378 genes were predicted targets of both miRNAs. GO or KEGG enrichment analysis of showed that the target genes of miR-200c and miR-141 were mainly in regulation of RNA polymerase II repressing transcription factor binding, and signaling receptor activity. Based on the data of DLBCL in the GEPIA2 database (http://GEPIA2.cancer-pku.cn/#index), 1321 target genes were differently expressed in DLBCL, of which 183 were target genes of both miR-200c and miR-141.

**Fig. 6:**
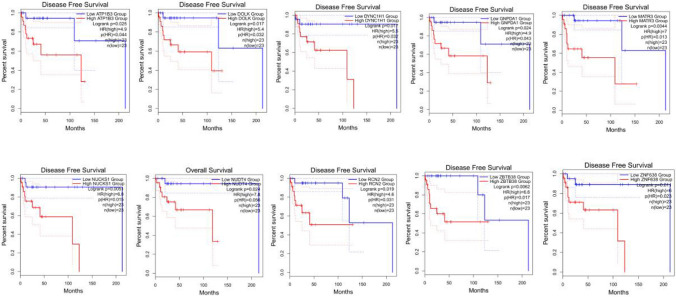
Relationships of *ATP1B3*, *DYNC1H1*, *MATR3*, *NUCKS1*, *ZNF638*, *NUDT4*, *RCN2*, *GNPDA1*, *ZBTB38*, and *DOLK* with survival in DLBCL

The intersection of the top 500 genes associated with OS and the top 500 genes associated with DFS among the aforementioned 1321 target genes illustrated that 24 of the target genes significantly affected patient survival. Based on the survival analysis results of the 24 genes and their corresponding hazard ratios, it was found that 10 of the 24 genes had a significant impact on OS or DFS (*ATP1B3*, *DYNC1H1*, *MATR3*, *NUCKS1*, *ZNF638*, *NUDT4*, *RCN2*, *GNPDA1*, *ZBTB38*, and *DOLK*). Relationships of the 10 genes with survival in DLBCL were shown in Fig. 6. Among these genes, *ZNF638* and *MATR3* were target genes of both miR-200c and miR-141. *NUCKS1*, *NUDT4*, *RCN2*, *GNPDA1*, and *ZBTB38* were target genes of only miR-200c, whereas *ATP1B3*, *DYNC1H1*, and *DOLK* were target genes of only miR-141.

## Discussion

PCNSL is a highly heterogeneous type of non-Hodgkin's lymphoma [25]. Early detection and treatment of relapsed PCNSL can improve treatment response and survival [25]. Patients with PCNSL who receive a standard first-line treatment regimen are expected to have better outcomes. However, even among patients treated with high-dose methotrexate, which carries a good prognosis, approximately 50% of patients experience relapse [8]. Importantly, clarification of the molecular mechanisms associated with poor outcomes is a pressing need. Multiple studies revealed that exosomal miRNA plays important roles in the progression and aggressiveness of solid tumors; in addition, increasing evidence suggests that exosomal miRNA plays the same important roles in lymphoma [26, 27]. To identify sensitive liquid biopsy biomarkers that might improve early diagnosis and predict the response to chemotherapy, we assessed a wider range of CSF exosomal miRNAs that could distinguish patients with PCNSL from controls and facilitate further studies on early relapse.

First, we analyzed the miRNAs of CSF-derived exosomes from patients with PCNSL. CSF exosomal miRNA-seq allowed us to directly compare the expression profiles of patients with PCNSL before and after chemotherapy. Compared with the findings in de novo PCNSL, the exosomal expression of miR-200c and miR-141 was enriched in PCNSL in remission after treatment. miR-200c and miR-141 are members of the miR-200 family, which also includes miR-200a, miR-200b, and miR-429. These miRNAs are involved in advanced stages of cancer progression, and they might act as activators or suppressors of tumorigenesis [28]. Via pathway analysis of potential mRNAs targets of miR-200 family members, we observed upregulation of epidermal growth factor receptor (EGFR), mitogen-activated protein kinase (MAPK), and phosphoinositide 3-kinase (PI3K)/protein kinase B (AKT) signaling. As previously reported, EGFR/MAPK signaling can inhibit the invasion and migration of more than one tumor of the brain [29, 30]. The PI3K/AKT signaling pathway is highly active in DLBCL, and it serves as an attractive target in lymphoma therapy [31, 32]. Whether the general dysregulated signaling pathway in CSF exosomes affects PCNSL metastases and drug resistance deserves further study.

We also used qRT-PCR to detect and validate the expression of miR-200c and miR-141 in CSF exosomes. The results of this study illustrated that the expression of miR-200c and miR-141 was significantly lower in patients with PCNSL than in patients without PCNSL. Interestingly, these two miRNAs were markedly upregulated in patients with PCNSL after chemotherapy, indicating their significant clinical value for monitoring chemotherapy efficacy. The involvement of miR-200c and miR-141 in cancer development, progression, and drug resistance regulation has been demonstrated [33]. The plasma levels of miR-141 are significantly higher in patients with DLBCL than in normal subjects [34]. Nevertheless, there are no reports about miR-200c as a predictor of therapeutic outcomes in patients with DLBCL. Conversely, evaluation of the relationships between the CSF exosomal miR-200c and the clinical parameters of our patients with PCNSL revealed that lower miR-200c levels were associated with higher CSF IL-10 levels. CSF IL-10 levels were higher in patients with PCNSL than in control subjects. IL-10 participates in tumor immune escape by inducing the activation of IL-10–dependent regulatory B cells [35]. These data imply that the differentially expressed of miR-200c and miR-141 in CSF exosomes interacts with tumor cells via different signaling molecules and activates or inhibits tumor growth in the initial and developmental stages of cancers.

MTX is currently a widely used antitumor agent, particularly for human leukemia. It is known that MTX transporter genes are regulated at the post transcriptional level by microRNAs [36]. Previously study found miR-323b that targets *ABCC4* associated with MTX plasma levels, which encodes for multi-drug resistance protein 4 (MRP4) [37]. In addition, miR-5189, miR-595, and miR-6083 that might affect *SLC46A1*, *SLC19A1*, and *SLCO1A2* MTX transport gene regulation and could affect MTX levels in patients with B-cell acute lymphoblastic leukemia [38]. Wulin Shan et al. confirmed that the expression of miR-200c was down-regulated in MTX-resistant A549 cells [39]. Nowadays miRNA involved in regulation of MTX transporter genes in PCNSL are not completely defined. Our results also indicated that miR-200c and miR-141 might modulate the sensitivity of DLBCL to MTX and inhibit tumors cells migration and invasion.

To our knowledge, this is the first study on the deep sequencing of miRNAs in CSF-derived exosomes from patients with PCNSL. Although miRNAs from CSF exosomes could reflect the characteristics of PCNSL from one aspect, they lack information on tumor cells from the primary lesion. Additionally, our study cohort was relatively small. A larger external cohort is needed to validate our results. Another caveat of our study was the lack of a functional assay to verify the miRNA target genes. In fact, we have observed that there were M2 tumor-associated macrophages (TAM) infiltrating in PCNSL tumor tissue. We hypothesized that miR-200c and miR-141 play an important role in macrophage reprogramming and polarization. Therefore, we will intend to elucidate the molecular mechanism of miR-200c and miR-141 participating in the TAM recruitment, the polarization of M2 TAM and regulation of PCNSL proliferation.

## Conclusions

Our findings revealed the characteristics of CSF-derived exosomal miRNAs in patients with PCNSL. We discovered that miR-200c and miR-141 might be involved in the onset and development of PCNSL. Our results will be useful for the development of more targeted therapies for patients with PCNSL. Furthermore, CSF exosomal miR-200c and miR-141 might serve as biomarkers for the early diagnosis of PCNSL and for monitoring the efficacy of chemotherapy.

### Electronic Supplementary Material

Below is the link to the electronic supplementary material

Supplementary Material 1

## Data Availability

All data generated or analyzed during this study are included in this published article and its Additional files. The information about experimental sessions and results are available from the corresponding author on reasonable request.
