# Supplementary material for: Exosomal miR-200c and miR-141 as cerebrospinal fluid biopsy biomarkers for the response to chemotherapy in primary central nervous system lymphoma
Source: Discov Oncol. 2023 Nov 16;14:205. doi: 10.1007/s12672-023-00812-1 (PMC10654293; doi:10.1007/s12672-023-00812-1)
Supplement: Supplementary file 1 — Supplementary Material 1 [file 12672_2023_812_MOESM1_ESM.docx]

**Supplementary Information**

Supplementary methods

**TEM**

Exosome solution (10 µL) was placed on a copper mesh and incubated at room temperature for 1 min. After washing with sterile distilled water, the exosomes were incubated with uranyl acetate solution for 1 min. The sample was then dried for 2 min under incandescent light. The copper mesh was observed and photographed under a transmission electron microscope (H-7650, Hitachi Ltd., Tokyo, Japan).

**NTA**

Vesicle suspensions with concentrations between 1 × 10^7^/mL and 1 × 10^9^/mL were examined using the ZetaView PMX 110 (Particle Metrix, Meerbusch, Germany) equipped with a 405-nm laser to determine the size and quantity of particles isolated. A 60-s video was taken with a frame rate of 30 frames/s, and particle movement was analyzed using NTA software (ZetaView 8.02.28).

**Library preparation and sequencing**

For small RNA libraries, 1–500 ng of RNA per sample were used as the input for RNA sample preparation. Sequencing libraries were generated using a QIAseq miRNA Library Kit (Qiagen, Frederick, MD, USA) following the manufacturer’s recommendations, and index codes were added to attribute sequences to each sample. Reverse transcription primers with unique molecular indices (UMIs) were introduced to analyze the quantification of miRNA expression during cDNA synthesis and PCR amplification. Finally, library quality was assessed using the Agilent Bioanalyzer 2100 and qPCR. The clustering of the index-coded samples was performed on the acBot Cluster Generation System using TruSeq PE Cluster Kitv3-cBot-HS (Illumina, San Diego, CA, USA) according to the manufacturer’s instructions. After cluster generation, the library preparations were sequenced on an Illumina NovaSeq 6000 platform, and paired-end reads were generated at EchoBiotech Co. Ltd.

**miRNA-seq data analysis and differential expression analysis**

Raw data of fastq format were initially processed using in-house perl scripts. In this step, clean data (clean reads) were obtained by removing reads containing adapters, reads containing ploy-N, and low-quality reads from raw data. At the same time, Q20, Q30, the GC content, and the sequence duplication level of the clean data were calculated. All downstream analyses were based on clean data with high quality. The Bowtie soft was used to align Clean Reads with Silva database, GtRNAdb database, Rfam database and Repbase database, respectively. Then the repeats and ncRNA, such as ribosomal RNA (rRNA), transfer RNA (tRNA), small nuclear RNA (snRNA), and small nucleolar RNA (snoRNA) were filtered. The remaining clean reads were used to detect known and new miRNAs predicted via comparisons with known miRNAs from miRbase and Human Genome (GRCh38), respectively. The expression matrix of quantified UMI counts of miRNAs was normalized to transcripts per million (TPM) and calculated as the relative log expression via the EdgeR package.

Differential expression analysis of the two groups was performed using the edgeR package with cutoffs of TPM > 10, P < 0.05, and absolute expression fold change > 1.55. qRT-PCR was performed to confirm the conformation. Volcano plots were generated to visualize the differentially expressed miRNAs using R package “ggplot2.”

Table S1. The primer sequences used for qRT-PCR

| Primers | Sequences |
| --- | --- |
| qPCR-TYR | GTGCAGGGTCCGAGGT |
| U6-RT | AACGCTTCACGAATTTGCGT |
| U6-S | CTCGCTTCGGCAGCACA |
| U6-A | AACGCTTCACGAATTTGCGT |
| U6 probe | AGAAGATTAGCATGGCCCCTGCGCA |
| miR-200c-3p-RT | GTCGTATCCAGTGCAGGGTCCGAGGTATTCGCACTGGATACGACTCCATC |
| miR-200c-3p-F1 | ACGCTAATACTGCCGGGTAAT |
| miR-200c-3p-P | TTCGCACTGGATACGACTCCATC |
| miR-141-3p-RT | GTCGTATCCAGTGCAGGGTCCGAGGTATTCGCACTGGATACGACCCATCT |
| miR-141-3p -F2 | TCGCTAACACTGTCTGGTAAAG |
| miR-141-3p -P | TTCGCACTGGATACGACCCATCT |


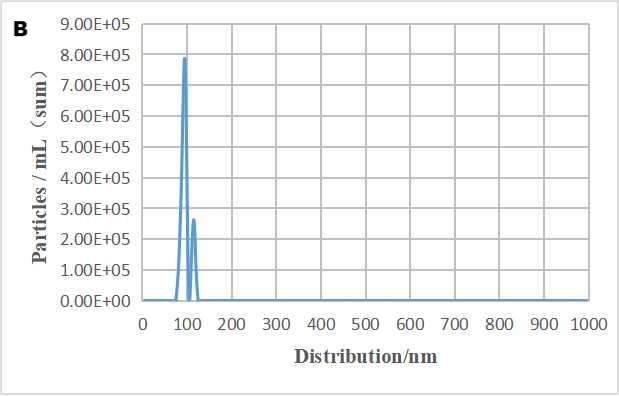


Figure S1. Characterization of the CSF exosomes. (A) TEM of the preparation from patients’ CSF revealing small vesicles of 100 nm (scale bars, 500 nm). (B) Size distribution analysis of CSF exosomes by NTA.

Table S2. Quality control metrics of CSF exosomal miRNA sequencing libraries

| Samples ID | Raw reads | Clean reads | ≥ Q30 bases (%) | miRNAs |
| --- | --- | --- | --- | --- |
| ID1 | 25566312 | 7880787 | 96.69 | 324 |
| ID2 | 22446096 | 8440224 | 96.89 | 387 |
| AT1 | 20232409 | 5257456 | 96.72 | 325 |
| AT2 | 21620360 | 6453587 | 96.59 | 311 |

ID, initial diagnosis; AT, after treatment

Table S3. Nine miRNAs differentially expressed between patients initially diagnosed with PCNSL and previously treated patients (P < 0.01)

| miRNA | Transcripts Per Kilobase Million | | | | Log2FC | P value |
| --- | --- | --- | --- | --- | --- | --- |
|  | ID1 | ID2 | AT1 | AT2 |  |  |
| miR-150-5p | 14285.39 | 28284.24 | 2258.59 | 1079.69 | -3.67 | 5.58E-04 |
| miR-200a-3p | 158.73 | 161.26 | 4189.85 | 2091.91 | 4.30 | 6.86E-04 |
| miR-141-3p | 204.08 | 161.26 | 5237.32 | 1979.44 | 4.30 | 8.60E-04 |
| miR-148a-3p | 2199.50 | 2418.83 | 17283.14 | 16442.85 | 2.87 | 1.78E-03 |
| miR-200c-3p | 158.73 | 64.50 | 12340.43 | 539.85 | 5.85 | 5.45E-03 |
| miR-660-5p | 45.35 | 215.01 | 0 | 0 | -16.99 | 2.88E-02 |
| Unconservative 1 7886 | 0 | 21.50 | 130.93 | 224.94 | 4.05 | 2.95E-02 |
| miR-20b-5p | 113.38 | 75.25 | 0 | 0 | -16.53 | 3.01E-02 |
| miR-532-3p | 45.35 | 150.51 | 0 | 0 | -16.58 | 3.68E-02 |

ID, initial diagnosis; AT, after treatment; FC, fold change
